# Tocilizumab and Baricitinib for Recovery From Acute Exacerbation of Combined Pulmonary Fibrosis and Emphysema Secondary to COVID-19 Infection: A Case Report

**DOI:** 10.7759/cureus.23411

**Published:** 2022-03-22

**Authors:** Yoshiro Kai, Masayuki Matsuda, Kentaro Suzuki, Takehito Kasamatsu, Akihiro Kajita, Kenji Uno, Shigeo Muro

**Affiliations:** 1 Department of Respiratory Medicine, Minami-Nara General Medical Center, Nara, JPN; 2 Department of Infectious Diseases, Minami-Nara General Medical Center, Nara, JPN; 3 Department of Respiratory Medicine, Nara Medical University, Nara, JPN

**Keywords:** acute respiratory failure, cpfe, covid-19, tocilizumab, baricitinib

## Abstract

Pneumonia secondary to coronavirus disease 2019 (COVID-19) is exacerbated by a disproportionate increase in the systemic inflammatory response and cytokine storm due to severe acute respiratory syndrome coronavirus 2 (SARS-CoV-2) infection. Herein, we report the successful treatment of severe COVID-19 pneumonia using a combination of tocilizumab and baricitinib in a patient with combined pulmonary fibrosis and emphysema (CPFE). A 67-year-old male with type 2 diabetes mellitus and CPFE presented with fever and dyspnea and was diagnosed with COVID-19. Upon admission, his respiratory failure was managed using high-flow nasal cannula (HFNC) therapy; however, despite treatment with remdesivir and systemic steroids, his respiratory failure continued to worsen. Therefore, baricitinib was administered from the ninth day of hospitalization for 14 days. Furthermore, his blood interleukin-6 (IL-6) levels showed an increase until day 13. Thus, tocilizumab was administered on the 13th day, which led to symptomatic improvement by day 18. The patient was discharged from our hospital on day 42. This case indicates that combination therapy with tocilizumab and baricitinib improves the efficacy of COVID-19 treatment in patients with comorbidities.

## Introduction

Severe acute respiratory syndrome coronavirus 2 (SARS-CoV-2) infection was first reported in December 2019; however, effective therapy for COVID-19-associated acute lung injury remains to be elucidated [1]. Tocilizumab, a humanized antihuman interleukin (IL)-6 receptor monoclonal antibody, is typically used to treat rheumatoid arthritis and cytokine release syndrome [2]. Baricitinib, an anti-Janus kinase (JAK) inhibitor, is an immunomodulator that has shown some promise in managing patients with severe COVID-19, and it is considered effective against COVID-19-induced cytokine storm and pneumonia [3]. Here, we describe the use of tocilizumab and baricitinib in a patient with combined pulmonary fibrosis and emphysema (CPFE) who developed acute respiratory failure due to COVID-19. In this case, this strategy averted the need for mechanical ventilation and facilitated the successful treatment of COVID-19.

## Case presentation

A 67-year-old male, under medication for type 2 diabetes mellitus, dyslipidemia, and CPFE, was admitted to our department with a one-day history of fever and muscular pain. Polymerase chain reaction test for COVID-19 infection via nasopharyngeal swab was positive. He presented with a pulse rate of 114 bpm, temperature of 38.1°C, blood pressure of 175/105 mmHg, and percutaneous oxygen saturation of 87% on 4 L/minute O2 administered via a nasal cannula. Clinical examination revealed bilateral fine end-inspiratory crackles at his lung bases. He was a former smoker (1 pack/day between ages 20 and 60 years) but had no history of asbestos exposure, bird rearing, or familial interstitial lung disease (ILD). He had been on long-term home oxygen therapy (4 L/minute O2 administered via nasal cannula) for chronic hypoxia due to CPFE for the last four years. His medical history was also notable for an acute exacerbation of CPFE one year ago, which was treated with intravenous methylprednisolone (1000 mg/day) for three days, followed by oral methylprednisolone 40 mg/day. Oral steroid intake was gradually tapered, and his maintenance regimen included oral prednisolone 5 mg/day and oral nintedanib 300 mg/day.

**Figure 1:**
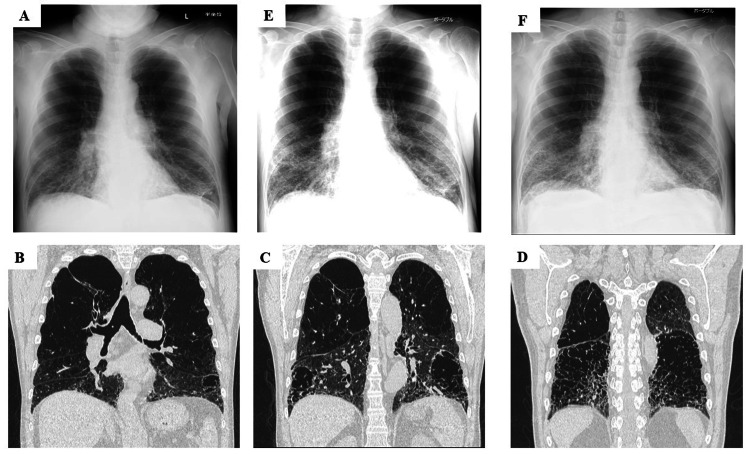
Chest radiographic images taken at different time points Chest X-ray (A) and computed tomography (B, C, and D) on admission show diffuse ground-glass shadows in bilateral lung fields. Diffuse ground-glass shadows on chest X-ray worsened by day 8 (E). Tocilizumab and baricitinib treatment improved findings on chest X-ray by day 25 (F).

**Figure 2:**
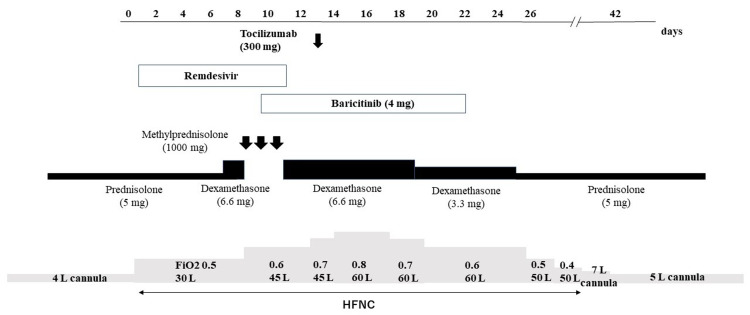
Clinical course Administration of tocilizumab and baricitinib improved oxygenation. HFNC, high-flow nasal cannula

Laboratory data upon admission revealed normal lactate dehydrogenase (LDH) (194 U/L) (Table 1) and slightly elevated C-reactive protein (CRP) (1.46 g/dL). Partial pressure of arterial oxygen (PaO2) declined to 52.3 Torr when oxygen was provided at the rate of 4 L/minute via nasal cannula. Chest X-ray revealed peripheral consolidation in the lower lung fields (Figure 1A), and chest computed tomography revealed bilateral ground-glass opacity (GGO) (Figure 1B-1D). The clinical course is shown in Figure 2. On admission, given his poor respiratory status, oxygen was provided via high-flow nasal cannula (HFNC) (fraction of inspiratory oxygen: 0.5, oxygen flow rate: 30 L). Remdesivir, an antiviral drug, was administered from day 1 to day 10. His respiratory status remained unaltered. Because an increase in CRP levels and radiological deterioration were observed, dexamethasone (6.6 mg/day) was added from day 6 onward. Additional methylprednisolone (1000 mg/day) was administered intravenously for three days from day 8. However, on day 8, because his respiratory status and chest X-ray image worsened (Figure 1E), oral baricitinib 4 mg/day was added from day 9 for two weeks. Baricitinib was just approved for COVID-19 treatment. He agreed on the use of baricitinib at this time. On day 13, serum IL-6 and LDH levels increased (Table 1), and intravenous tocilizumab 300 mg/day was administered only on day 13. From day 17, his respiratory conditions, such as dyspnea, tachypnea, and oxygenation, gradually improved, and serum IL-6 level decreased from 489 pg/mL on day 13 to 144 pg/mL on day 17; LDH level also decreased in parallel from 295 U/L on day 13 to 242 U/L on day 17 (Table 1). Chest X-ray image finally improved on day 25 (Figure 1F); however, KL-6 levels remained unaltered (Table 1). His condition stabilized with the administration of 5 L oxygen by nasal cannula, and on day 40, IL-6 and LDH levels significantly decreased to 69 pg/mL and 180 U/L, respectively (Table 1). He was discharged on day 42 after rehabilitation and with instructions for home oxygen therapy. Follow-up chest X-ray images after discharge confirmed the absence of recurrence.

**Table 1:** Change in IL-6, KL-6, and LDH Laboratory data upon admission revealed normal lactate dehydrogenase (LDH) (194 U/L). On day 13, serum IL-6 and LDH levels increased. Baricitinib was administered from the ninth day of hospitalization for 14 days. Tocilizumab was administered on the 13th day. From day 17, his respiratory conditions gradually improved, and serum IL-6 level decreased from 489 pg/mL on day 13 to 144 pg/mL on day 17; LDH level also decreased in parallel from 295 U/L on day 13 to 242 U/L on day 17. On day 40, IL-6 and LDH levels significantly decreased to 69 pg/mL and 180 U/L, respectively. IL-6, interleukin-6; KL-6, sialylated carbohydrate antigen; LDH, lactate dehydrogenase

	Day 2	Day 13	Day 17	Day 20	Day 40
IL-6 (<7 pg/mL)	108	489	144	113	<69
KL-6 (<500 U/mL)	511	811	862	897	909
LDH (124–222 U/L)	194	295	242	221	180

## Discussion

Although COVID-19-associated cytokine storm is thought to play a role in the exacerbation of COVID-19 pneumonia, few treatment regimens can control it. In the present case, tocilizumab and baricitinib improved both dyspnea and tachypnea, which ensured not only adequate oxygenation despite acute respiratory failure in CPFE but also a favorable outcome without the use of mechanical ventilation. Chronic lung disease is a reported risk factor for COVID-19, and CPFE, defined as a syndrome, is characterized by the coexistence of emphysema in superior pulmonary locations and predominant fibrosis of the lower lobes. Chronic obstructive pulmonary disease and smoking in patients with COVID-19 are associated with increased risk of severe disease and mortality [4], and comorbid ILD is a risk factor for poor outcomes in patients with COVID-19 [5]. IL-6 plays a critical role in the cytokine storm as it promotes helper T cell response but inhibits regulatory T cell function [1,2]; thus, tocilizumab, a recombinant anti-IL-6 monoclonal antibody, improves outcomes of patients with COVID-19 pneumonia and cytokine storm [6]. Baricitinib, an inhibitor of the JAK-signal transducer and activator of transcription pathway, affects the production of inflammatory cytokines that contribute to the cytokine storm, such as IL-1β, IL-6, tumor necrosis factor-α, and IL-8 [7]. Notably, baricitinib has shown promise in managing symptomatic patients with COVID-19, and it is the first immunomodulatory drug to reduce COVID-19 mortality in a placebo-controlled trial [3]. Initially, our patient was prescribed only an antiviral drug (remdesivir) and dexamethasone, and expansion of opacities in bilateral lower lung fields and greater oxygen requirement via HFNC were paralleled by an increase in serum IL-6 levels. In this case, COVID-19-triggered acute exacerbation of interstitial pneumonia (AE-IP) was considered. Enhancement of sound of fine end-inspiratory crackles and elevation of KL-6 and LDH levels supported this finding. On day 8, the computed tomography finding of AE-IP included widespread GGO in the bilateral lower lung fields. We decided to initiate methylprednisolone pulse therapy based on the possibility of AE-IP. However, methylprednisolone pulse therapy was insufficient and did not aid improvement. The administration of tocilizumab and baricitinib alleviated worsening symptoms, i.e., improved oxygenation and reduced serum IL-6 levels. A recent report has stated that the treatment of interstitial pneumonia secondary to COVID-19 with tocilizumab and baricitinib did not cause serious side effects, indicating that this combination may be administered early in patients with COVID-19 and impaired arterial oxygen (PaO2) or fraction of inspiratory oxygen [8]. The suppression of excessive inflammation through the timely administration of tocilizumab and baricitinib in the early stage of COVID-19-triggered AE-IP may have effectively prevented invasive positive pressure ventilation in the present case. Thus, although the effectiveness of anti-inflammatory treatment against cytokines in patients with COVID-19 remains unclear, combination therapy with tocilizumab and baricitinib may be useful for avoiding intubation in high-risk patients. Further studies are needed to determine adequate timing of therapy administration based on clinical parameters such as IL-6.

## Conclusions

This case report shows that tocilizumab and baricitinib improved both dyspnea and tachypnea, which ensured not only adequate oxygenation despite acute respiratory failure due to CPFE but also a favorable outcome without the use of mechanical ventilation. Combination therapy with tocilizumab and baricitinib is a viable option for the treatment of COVID-19 in patients with comorbidities. The effectiveness of this therapy for cytokine storms needs further research.
